# Multifaced Roles of the Urokinase System in the Regulation of Stem Cell Niches

**Published:** 2018

**Authors:** K. V. Dergilev, V. V. Stepanova, I. B. Beloglazova, Z. I. Tsokolayev, E. V. Parfenova

**Affiliations:** Laboratory of Angiogenesis, National Medical Research Center of Cardiology, 3rd Cherepkovskaya Str., 15a, Moscow, 121552, Russia; Department of Pathology and Laboratory Medicine, University of Pennsylvania Perelman School of Medicine, Philadelphia, USA; Laboratory of Post-Genomic Technologies in Medicine, Faculty of Fundamental Medicine, Moscow State University, Lomonosovsky Ave., 27-1, Moscow, 119991, Russia

**Keywords:** urokinase, urokinase receptor, plasminogen activator inhibitors, regeneration, stem cells, cell niches

## Abstract

Proliferation, subsequent migration to the damaged area, differentiation into appropriate cell types, and/or secretion of biologically active molecules and extracellular vesicles are important processes that underlie the involvement of stem/progenitor cells in the repair and regeneration of tissues and organs. All these functions are regulated through the interaction between stem cells and the microenvironment in the tissue cell niches that control these processes through direct cell-cell interactions, production of the extracellular matrix, release of extracellular vesicles, and secretion of growth factors, cytokines, chemokines, and proteases. One of the most important proteolytic systems involved in the regulation of cell migration and proliferation is the urokinase system represented by the urokinase plasminogen activator (uPA, urokinase), its receptor (uPAR), and inhibitors. This review addresses the issues of urokinase system involvement in the regulation of stem cell niches in various tissues and analyzes the possible effects of this system on the signaling pathways responsible for the proliferation, programmed cell death, phenotype modulation, and migration properties of stem cells.

## INTRODUCTION

Currently, stem cells (SCs) are considered as an important regulator of cellular homeostasis and a component of the regeneration/repair of all body tissues. SCs have already been used in medical practice; however, production of biomedical products with certain properties remains an unsolved problem due to the complex, not fully understood pathways of regulation which underlie their unique properties. Regulation of SC functions in tissues involves a certain microenvironment that forms specific structures called “cell niches” [1, 2]. This microenvironment originates from interactions between stem cells and neighboring differentiated cells, as well as components of the extracellular matrix (ECM) due to the activation/inhibition of various signaling pathways (Notch, Wnt, TGF-β, Sonic Hedgehog, etc.) through direct cell-cell interactions, release of extracellular vesicles, and secretion of growth factors, cytokines, chemokines, and various proteases [3]. An important component of this complex regulation is the urokinase system represented by urokinase (also known as urokinase-type plasminogen activator (uPA), its receptor (uPAR/CD87), and two of its inhibitors (PAI-1 and PAI-2). The uniqueness of this system is related to the urokinase receptor anchored to the cell membrane by glycosylphosphatidylinositol, which enables the receptor to move in the membrane bilayer and locally concentrate the proteolytic activity of urokinase in the direction of cell movement. The urokinase-triggered cascade of proteolytic reactions, including the local formation of plasmin and activation of matrix metalloproteinases, promotes degradation of the ECM along a path of a moving cell, activation of growth factors, and release of the growth factors sequestered in the matrix [4-7]. However, in addition to the activation of extracellular proteolysis, most cellular responses modulated by the urokinase system require transmembrane signaling. This signaling is mediated by the interaction between components of this system and a variety of extracellular and intracellular proteins and membrane receptors that transmit signals to the intracellular pathways that regulate various cellular functions. The urokinase system components are present in the niches of bone marrow stem cells [8], striated muscles [9], neural cells [10], and tumor cells [11]. They are involved in the regulation of important biological processes, such as inflammation, angiogenesis, myogenesis, remodeling of extracellular matrix proteins, metastasis, and tumor growth. This review discusses potential ways for regulating stem cell functions by the urokinase system through extracellular matrix remodeling and interaction with the signaling pathways responsible for the regulation of division, programmed cell death, and modulation of the phenotype and cell motility, which is important in the development of approaches to directed influence on their properties.

## UROKINASE SYSTEM: STRUCTURE AND FUNCTIONS

**Fig. 1:**
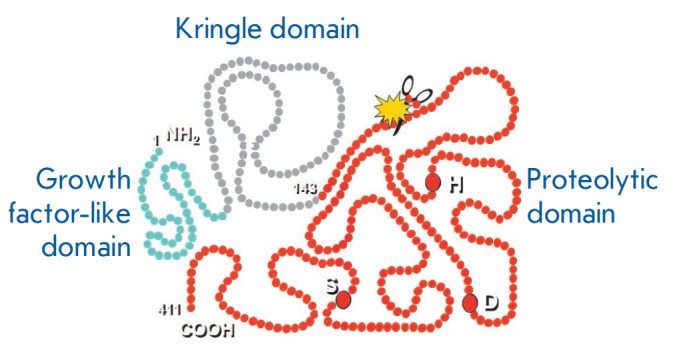
Schematic representation of the urokinase structure

Urokinase is an extracellular serine protease with narrow substrate specificity which is involved in the conversion of plasminogen to plasmin. In humans, urokinase is secreted by various cell types: monocytes/ macrophages [12, 13], tumor cells [14-16], fibroblasts [17, 18], smooth muscle cells [19, 20], and endothelial cells [21, 22]. Urokinase consists of 411 amino acid residues (molecular weight of 53 kDa) [23] and is secreted by cells as a single-chain protein (sc-uPA) comprising three domains: a N-terminal growth factor-like domain (GFD) structurally homologous to the epidermal growth factor (residues 9–45), a kringle domain (KD, residues 45–134), and a C-terminal proteolytic domain (PD, residues 144–411). The growth factor-like domain function is high affinity interaction with the urokinase receptor on the cell surface [24]. The proteolytic domain converts plasminogen into plasmin and activates some growth factors and matrix metalloproteinases [25]. The function of the kringle domain is not yet fully understood; however, the domain is believed to be involved in the stimulation of cell migration under the action of urokinase [26], stabilize the interaction between urokinase and the receptor [27], and participate in the transport of urokinase into the nucleus [28] (*Fig. 1*).

**Fig. 2:**
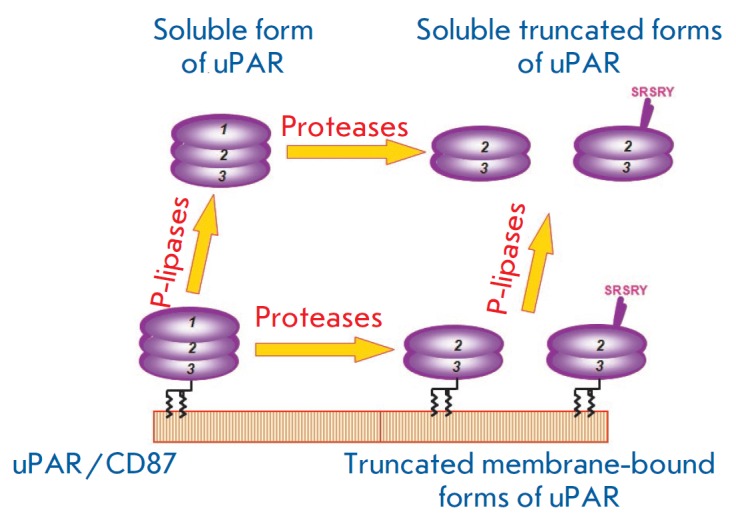
Action of proteases and phospholipases leads to formation of truncated membrane-bound and soluble forms of the urokinase receptor

The urokinase receptor uPAR/CD87 was first identified as a urokinase-type plasminogen activator receptor on the surface of human monocytes [29]. uPAR was also detected on endothelial cells [30], neutrophils [31], smooth muscle cells [32], placental trophoblast cells [33], and also on the cells of various tumor lines [34-37]. uPAR/CD87 is overexpressed by blood cells during inflammation [38, 39]. uPAR belongs to the Ly-6 family [40] and is a single-chain, highly glycosylated protein [41] anchored to the cell membrane by glycosylphosphatidylinositol covalently bound to the third, C-terminal domain of the receptor [42]. uPAR has a molecular weight of 55–60 kDa and consists of 313 amino acid residues that form three structurally homologous domains [43]. The first domain of the receptor plays a major role in the binding to urokinase and interacts with its growth factor-like domain. According to crystallography data, the ligand-bound urokinase receptor occurs in a more compact state, because the first and third domains of the receptor come in close proximity during its interaction with urokinase. One of the important processes regulating the uPAR function is proteolytic cleavage between the first and second domains (*Fig. 2*) by proteases such as plasmin, matrix metalloproteinases, and urokinase itself [44, 45]. After cleavage, uPAR loses its ability to bind urokinase, but it acquires the ability to regulate cell migration independently of urokinase [46]. Both the full-length and cleaved (c-uPAR) forms of the urokinase receptor can be removed from the membrane surface by proteases or phospholipase C specific to glycosylphosphatidylinositol [47-52]. This process results in soluble full-length (su-uPAR) and cleaved (su-c-uPAR) forms of the receptor, which circulate in the blood plasma and serve as markers of some inflammatory or immunological diseases. It is important to note that the soluble cleaved urokinase receptor is a strong chemoattractant for cells (neutrophils, monocytes, macrophages) expressing receptors for the bacterial N-formyl-methionyl-leucyl-phenylanilanne (fMLP) peptide [53, 54].

A high level of urokinase proteolytic activity may be detrimental to cells. To regulate the level of extracellular proteolysis, cells synthesize specific protein inhibitors of plasminogen activators – PAI-1, PAI-2, protease nexin-1, and protein C inhibitor [55-58]. They belong to a group of arginine-serpin inhibitors. They mimic the substrate during interaction with a double-chain form of the enzyme, which results in a 1 : 1 stable covalent enzyme–inhibitor complex and enzyme inactivation [59]. The interaction with single-chain urokinase does not lead to a covalent complex. PAI-1 is a 45–50 kDa single-chain glycoprotein. After secretion, PAI-1 is rapidly inactivated due to conformational rearrangements and becomes unable to bind to urokinase. Activation of the inhibitor requires the interaction of an inactive PAI-1 molecule with physiological cofactors – the extracellular matrix protein vitronectin or heparin [60]. Matrix-bound PAI-1, unlike its free form, can remain active for a long time [61]. Active PAI-1 interacts with both free and receptor-bound urokinase, inhibiting the pericellular proteolysis process [62]. Single-chain urokinase has low proteolytic activity and can also bind PAI-1, but at a much lower rate [63]. The PAI-1 activity can be regulated in several ways. Urokinase is able to cleave and inactivate PAI-1 [64]. In addition, binding of PAI-1 to uPA/uPAR leads to a ternary complex that is immediately internalized by cells [65, 66]. This process is triggered by the interaction between the ternary complex and endocytic receptors from the low-density lipoprotein receptor family. Urokinase and PAI-1 are degraded in the lysosomes, and the uPAR and endocytic receptor return to the cell surface, thereby initiating intracellular signaling and cytoskeleton rearrangement. Therefore, along with the ability to regulate proteolytic activity, PAI-1 is involved in the regulation of cell migration and adhesion.

The PAI-2 urokinase inhibitor is a 47 kDa single-chain glycoprotein [67]. Its ability to inhibit urokinase is much lower than that of PAI-1. For example, the constant for association of receptor-bound urokinase with PAI-1 is 15-fold greater than that with PAI-2 [63]. For a long time, inhibition of urokinase had been believed to be the main function of PAI-2. However, only a small fraction of the newly synthesized inhibitor is found to be secreted as a glycosylated polypeptide into the extracellular space [68]. The main fraction remains inside cells and protects them from the apoptosis induced by the tumor necrosis factor-α (TNF-α) [69, 70], as well as regulates the level of interferon-α/β secretion [71]. The secreted form of PAI-2 is involved in the regulation of fibrinolysis and tissue remodeling. The cytosolic form of PAI-2 plays an important role in the intracellular proteolysis involved in the regulation of apoptosis and inflammation.

## UROKINASE SYSTEM AND HEMATOPOIETIC BONE MARROW STEM CELLS

The bone marrow contains a population of hematopoietic stem cells (HSCs) capable of self-renewal and differentiation into all blood cells and some other cell types. In the bone marrow, HSCs express uPAR on their surface and are localized in cell niches that are mainly represented by osteoblasts, endothelial cells, and mesenchymal stem cells [72, 73]. These cells are poorly differentiated and characterized by a low level of proliferation/apoptosis due to cell cycle arrest in the G0/G1 phase. However, in *uPAR*-deficient mice (Plaur–/– mice), HSCs actively enter the cell cycle, differentiate, and enter the systemic circulation, which reduces their poorly differentiated pool and indicates the role of the urokinase receptor in maintaining the low-differentiated state of HSCs [74]. In addition, uPAR controls post-transplant survival of HSCs and the efficiency of hematopoietic recovery [74]. HSCs obtained from transgenic uPAR–/– mice and transplanted to wild-type splenectomized mice after radiation exposure (9.5 Gy) had reduced indicators of bone marrow integration and survival for a 2-week follow-up period compared to those of wild-type mouse HSCs. One of the potential molecular mechanisms of these effects may be the interaction of uPAR with integrins, in particular with α4β1-integrin that regulates migration and adhesion of HSCs to fibronectin and VCAM-1 during their homing and engraftment in the bone marrow [74-78]. The function of α4β1-integrin is known to depend on intact uPAR, because only the intact urokinase receptor interacts with integrins [79, 80]. Proteolytic cleavage of uPAR with removal of the D1 domain reduces α4β1-mediated cell adhesion [81]. Transgenic mice deficient in the urokinase receptor are characterized by impaired α4β1-integrin-mediated adhesion of HSCs in the bone marrow, which probably leads to disruption of their integration into the bone marrow tissue. Soluble forms of the urokinase receptor (s-uPAR) may play some role in HSC release from the bone marrow; the level of receptors significantly increases in blood plasma during mobilization of HSCs with the granulocyte colony-stimulating factor (G-CSF) [82, 83]. s-uPAR may facilitate migration of HSCs into the bloodstream, either directly or indirectly, by suppressing the activity of the CXCR4 receptor that is responsible for keeping cells in the bone marrow niche. *In vivo *experiments demonstrated that peptides developed on the basis of a cleaved s-c-uPAR form were able to induce the release of mouse CD34+ HSCs from bone marrow depots as efficiently as G-CSF [82].

Therefore, the urokinase receptor both maintains HSCs at rest in the bone marrow niche and regulates their release from the niche, probably, through several mechanisms, including interaction with integrins and direct chemotactic action.

## UROKINASE SYSTEM AND ENDOTHELIAL PROGENITOR CELLS

Pathogenesis of many cardiovascular diseases is associated with dysfunction and damage to the vascular wall endothelial layer that plays an important role in the regulation of the cardiovascular system function. Circulating endothelial progenitor cells (EPCs) released from bone marrow niches provide endothelial layer repair and postnatal vasculogenesis [84].

Damage to the vessel activates synthesis and secretion of a wide range of cytokines and chemokines (VEGF, IGF2, MCP-1, IL-8, bradykinin, MIF, SDF-1, etc.) that create a gradient inside the vascular wall and promote EPC homing to the damaged area via the adhesion and transendothelial migration mechanisms. The urokinase system is known to be involved in the regulation of angioarteriogenesis in ischemia and inflammation [85-87], in particular via regulation of directed migration of EPCs [88, 89] expressing high levels of uPA and uPAR [90]. In this case, in non-stimulated EPCs, the urokinase receptor is localized in lipid rafts and absent in caveolae; however, stimulation by VEGF causes increased expression of caveolin-1 and uPAR, assembly of caveolae, and uPAR internalization in EPCs [91]. Impairment of caveolae assembly in EPCs caused by methyl beta-cyclodextrin (β-MCD) or inhibition of caveolin-1 does not cause redistribution of uPAR on the cell membrane, while suppression of uPAR expression disrupts the normal organization of caveolae. These data suggest that uPAR may be an organizer of the assembly of caveolar rafts in EPCs, which underlies the behavior of these cells in the vascular wall [92]. For example, caveolin-dependent ERK1/2 phosphorylation stimulated by VEGF is the initiating event in migration/ differentiation of EPCs, and the caveolae integrity affects the angiogenic properties of EPCs [93]. VEGF increases expression of caveolin-1 and uPAR in EPCs and triggers redistribution of uPAR in caveolae, which increases invasion of EPCs and promotes capillary morphogenesis. Suppression of uPAR expression by antisense oligonucleotides disrupts caveolae formation and inhibits EPC invasion and capillary genesis. [93]. Thus, the formation of caveolar uPAR is considered a critical step in implementation of the angiogenic properties of EPCs. Secretion of uPA and the precursor of matrix metalloproteinase-2 (pro- MMP-2) is also increased in EPCs stimulated with VEGF or TNF-α [93], and inhibition of uPA or uPAR by monoclonal antibodies significantly reduces proliferation, migration, and formation of capillary-like structures by these cells *in vitro *[93, 94]. Recently, autophagy was shown to play a certain role in the regulation of EPC migration [95], which regulates, via the mTOR-P70S6K signaling pathway, expression of uPA and matrix metalloproteinases that degrade extracellular matrix proteins, which is necessary for migration of EPCs to the damaged area. Therefore, the existing data indicate the crucial role of urokinase and its receptor in providing homing into the injured vessel and the angiogenic properties of circulating endothelial progenitor cells.

## UROKINASE SYSTEM AND PROGENITOR CELLS OF STRIATED MUSCLE TISSUE

Satellite cells (SCs) form a stable self-renewing pool in the skeletal muscles of an adult organism. As revealed by electron microscopy more than four decades ago, striated muscle stem cells are mononuclear cells located between the muscle fiber sarcolemma and the basal lamina surrounding the fiber [96]. This anatomical location acts as the basis of a cell niche where satellite cells can be maintained at rest or activated, divide, and differentiate in response to external stimuli associated with muscle growth and recovery. Activated SCs undergo division and give rise to myogenic progenitor cells – skeletal myoblasts [97]. Myoblasts begin to express myogenic transcription factors, such as MyoD, Myf5, MRF4, myogenin, and other muscle proteins, secrete uPA and PAI-1, express uPAR on the surface, and fuse to form muscle tubes that are the future muscle fibers [98, 99]. The urokinase system is involved in the regeneration of striated muscles through regulation of the functions of SCs and skeletal myoblasts. Binding of uPA to the receptor was shown to be necessary to initiate migration of SCs, their differentiation, and fusion with pre-existing myotubes. Blockade of this binding with antibodies inhibits migration of cultured G8-1 myoblasts and suppresses their ability for myogenic differentiation [100]. The latter may be due to suppressed expression of myogenin and MyoD, which occurs when binding of uPA to uPAR is inhibited [101].

Skeletal muscle regeneration is regulated by a balance between uPA and PAI-1, which may affect regeneration through several mechanisms, including triggering of intracellular signaling upon binding of urokinase to the receptor [99] and modulation of the effects of growth factors, in particular, FGF-2 [102]. Also, uPA is necessary for myoblast fusion when uPA expression in these cells increases manifold. Antibodies blocking the catalytic activity of uPA or the interaction between uPA and uPAR completely inhibit fusion and muscle tube formation [103, 104]. Therefore, uPA regulates proliferation, migration, and fusion of myoblasts. The mechanisms underlying this regulation cannot be explained solely by the proteolytic function of uPA and require further investigation.

## UROKINASE SYSTEM AND MESENCHYMAL STEM CELLS

Mesenchymal stem cells (MSCs) are found in almost all organs and tissues. Together with extracellular matrix proteins, MSCs form the microenvironment of resident stem cells in tissue cell niches [105]. They regulate tissue repair, modulating the properties of stem and immune cells and their homing due to secretion of a wide range of biologically active factors and release of the extracellular vesicles that transfer not only protein factors, but also regulatory miRNAs to recipient cells [106]. One of the most important properties of MSCs is their ability to stimulate the angiogenic behavior of endothelial cells (ECs) both through paracrine effects and through direct contacts in the vascular cell niche [107, 108]. In most tissues, MSCs are located in the vascular wall in the peri-endothelial and supra-adventitial compartments [109]. Peri-endothelial MSCs are able, through the basement membrane pores, to interact directly with endothelial cells, regulating their functions through direct contacts and secretory mechanisms. A definite role in this regulation is played by the urokinase system. MSCs isolated from the bone marrow and adipose tissue express and secrete all urokinase system components: uPA, uPAR, and PAI-1 [110, 111]. However, depending on the tissue origin of MSCs, their role in ECM remodeling during vascularization is different. A number of studies, including ours, have demonstrated that bone marrow and adipose tissue MSCs co-cultured with endothelial cells stimulate them to form tubular structures [111, 112], through different proteolytic systems. Bone marrow MSCs remodel the matrix through membrane-bound metalloproteinases during angiogenesis, and adipose tissue MSCs (AT-MSCs) remodel the matrix through activation of plasminogen by urokinase [111, 112]. Our *in vitro *experiments demonstrated that in the absence of exogenous ECM ECs need direct contact with MSCs to stimulate the formation of vascular structures. We also found a significant increase in the expression of the urokinase receptor on the surface of ECs co-cultured with AT-MSCs. The latter was found to be crucial for MSC-stimulated angiogenesis, because uPAR inhibitory antibodies dose-dependently inhibited formation of the capillary structures [111]. Other components of the urokinase system also played a significant role in the regulation of the angiogenic behavior of endothelial cells by mesenchymal stem cells, because inhibitors of urokinase system components (amiloride, LRP antagonist RAP protein) also inhibited MSC-stimulated angiogenesis [113]. These results suggest that in the vascular cell niche of adipose tissue the urokinase system plays an important role in the regulation of the angiogenic behavior of endothelial cells AT-MSCs. In addition, an important role in the formation of the vascular network, especially during its stabilization, is played by pericytes that are considered as vascular MSCs [114]. The urokinase system regulates directional migration of vascular mural cells [115, 116] and MSCs. In model *in vitro *experiments, uPA enhanced spontaneous migration of MSCs through induction of secretion of matrix metalloproteinase 9 by MSCs, and also mediated migration in response to PDGF-BB, because blockade of the interaction between uPA and uPAR antibodies completely inhibited PDGF-BB-induced MSC migration [117]. In addition, the uPA/uPAR system is absolutely necessary for intracellular signaling triggered by PDGF-AB to induce bone marrow and adipose tissue MSC migration [118], which indicates the important role of this system in the regulation of the directional movement of MSCs necessary for their participation both in vessel growth and in other physiological and pathological processes [109]. Another important effect of urokinase system activation is regulation of MSC differentiation. Intracellular signals from uPAR were shown to regulate adipogenic differentiation of MSC [119] through PI3K/Akt pathway activation, and their osteogenic differentiation [120] through the NF-kB-mediated mechanisms.

An important mechanism that regulates the properties of cells in tissue niches is their interaction with extracellular matrix proteins. Synthesizing and remodeling the matrix through proteolytic mechanisms, MSCs are able to regulate cell functions in the tissue niche and their own functions. These effects are explained by a change in the matrix density due to matrix remodeling by MSCs, which is important in determining the direction of differentiation [121]. It may be supposed that by remodeling the extracellular matrix in tissue niches, MSCs can regulate the differentiation properties of resident stem cells and that this regulation is mediated by signals that affect urokinase receptor expression in MSCs [73]. In addition, MSCs secrete urokinase that triggers a proteolytic cascade on the cell surface, which promotes the release of growth factors sequestered in the matrix surrounding cells and contributing to the regulation of the functions of both MSCs and other cells of the microenvironment. Therefore, the urokinase system is involved, through different mechanisms, in the regulation of the functions of MSCs and other cells in tissue cell niches and may be considered as a promising target for effects on these cells.

## UROKINASE SYSTEM AND TUMOR STEM CELLS

Studies in recent years have demonstrated that a population of tumor stem cells (TSCs) residing in the tumor tissue are responsible for initiation, spread (metastasis), and recurrence of tumors. TSCs were first found in the bone marrow in acute myeloid leukemia [122] and later in most solid malignant tumors of the ovaries [123], prostate [124], pancreas [125], large intestine [126], brain [127], etc. TSCs possess the main features of stem cells: resistance to radiotherapy and chemotherapy, the ability to quickly form the main populations of tumor cells and restore the cellular microenvironment, even after treatment. The role of the urokinase system in the development and metastasis of tumors has been explored for several decades, but there are only a few studies on tumor stem cells. According to the available data, the urokinase system may be considered as an important regulator of the state and development of TSCs. For example, plasmid overexpression of uPAR in human breast cancer MCF-7 and MDA-MB-468 cell lines caused the formation of TSCs with a characteristic immunophenotype CD24low/CD44high and containing stem phenotype markers – integrin β1/ CD29 and α6/CD49f [128]. A suspension of these cells transplanted into adipose tissue of the mammary gland of immunodeficient SCID mice resulted in pronounced integration of the graft into the tissues of the recipient animal and promoted a higher rate of primary tumor foci with a larger size than upon transplantation of cells transfected with control “empty” plasmids [128]. This indicates involvement of uPAR in the formation of the stem phenotype of tumor cells. Another mechanism for the regulation of TSC plasticity, which involves uPAR, is activation of the epithelial-mesenchymal transition (EMT). The results of numerous studies have confirmed that triggering of the EMT program in epithelial TSCs facilitates the mesenchymal phenotype in TSCs and increased expression of the stem phenotype markers contributing to the initiation of tumor development and metastasis [129-131]. Under hypoxic conditions, uPAR contributes to the initiation of EMT in a culture of human breast cancer MDA-MB-468 cells with an epithelial phenotype due to activation of different signaling mechanisms, including ERK1/2, PI3K/Akt, Src, and Rac1 [132, 133] . Preservation of the acquired mesenchymal phenotype of TSCs requires a high level of uPAR expression and is completely reversible upon suppression of uPAR expression, inhibition of the uPA–uPAR interaction, and blockade of PI3K, Src, and ERK1/2 signaling [132, 133]. Formed TSCs expressing uPAR can occur in tissues at rest (in the G0/G1 phase) for a long time, and proliferation/ growth of the dormant tumor can happen after many years. Another mechanism for the involvement of the urokinase system in the development of tumors is direct or plasmin-mediated activation of mitogens. For example, urokinase activates HGF that is secreted by fibroblasts as a single-chain biologically inactive precursor and accumulates in the extracellular matrix. Cleavage of HGF by urokinase produces an active protein heterodimer [134] that is a mitogen activating the proliferation of many cells, including TSCs. Other pro-mitogenic factors released from the matrix and activated by urokinase are FGF-2, VEGF189, IGF-1, and TGF-β [135-138]. The activity of uPA/uPAR in TSCs is regulated by plasminogen activator inhibitors – PAI-1/PAI-2 [139, 140]. However, their effect on TSCs is associated not only with the ability to inhibit the urokinase activity, but also with the ability to interact with vitronectin responsible for keeping cells in tumor niches. Vitronectin-bound PAI-1 reduces the interaction of vitronectin with integrins on the surface of TSCs and, thereby, promotes the release of TSCs from tumor niches, regulating their adhesion and migration.

Several years ago, we found a fundamentally new signaling pathway by which urokinase regulates acquisition of the stem phenotype by tumor cells and their resistance to cytotoxic agents. In particular, we demonstrated for the first time that urokinase is transported to the nucleus [28], where it binds to the transcription factors (HOXA5, HHEX, Lhx-2) involved in the regulation of the stem phenotype and survival of tumor [141] and endothelial [28] cells. Using fluorescent immunohistochemistry, we identified the localization of urokinase in the nuclei of tumor cells and in endothelial cells associated with the tumor [142]. The mechanism of uPA transport to the nucleus has not been fully studied, but we have demonstrated that the kringle domain of urokinase is necessary for the transport of urokinase to the nucleus, and we have also identified the nucleolin protein (Nuc1) that, binding to the kringle domain, is involved in the transport of urokinase to the nucleus [28]. Nucleolin, despite its preferential localization in the nucleus and nucleoli, is able to circulate between the cell membrane, cytoplasm, and nucleus and bind to different classes of proteins. In particular, it is involved in the transport of several secreted proteins, such as FGF-1, FGF-2, midkine, and laminin [143]. Nucleolin is recognized as one of the promising targets for anticancer therapy [144], and inhibition of urokinase transport into the nucleus may be one of the mechanisms of this effect [28]. Our data indicate that the urokinase receptor inhibits urokinase transport to the nucleus, retaining urokinase on the cell surface (V. Stepanova, unpublished data). We suppose that in tumor stem cells, where the urokinase level is significantly increased [142], urokinase is transported mainly to the nucleus, which is facilitated by removal of the first domain or the full-length urokinase receptor from the surface of tumor cells by proteases or shedding of the full-length uPAR by PI-PLC [47-52].

Further studies should provide answers to the following questions: 1) what form of the urokinase receptor (full-length or cleaved between the first and second domains) prevails on the surface of tumor cells that have a predominantly stem phenotype; 2) whether the rate of urokinase receptor removal from the surface of TSCs is increased; 3) whether the cells that have a predominantly stem phenotype have increased nuclear acummulation of uPA? These studies, in our opinion, will expand our understanding of the role of the urokinase system in the regulation of tumor stem cell functioning and define targets and ways to reduce their resistance and induce apoptosis.

## THE ROLE OF FIBRINOLYTIC SYSTEM COMPONENTS IN REGULATION OF HEART STEM/PROGENITOR CELL FUNCTIONS

The role of urokinase in the regulation of heart stem/ progenitor cell functions has been studied only in the most recent years. This system, as in tumor stem cells, was shown to be capable of controlling the epithelial– mesenchymal transition [145, 146] that produces the multipotent epicardial progenitor cells that represent some of the subtypes of the resident heart progenitor cells involved in regenerative processes through differentiation into blood vessel and myocardial cells and paracrine secretion of growth factors, cytokines, and exosomes [147-150]. There are only a few publications devoted to the role of the urokinase system in the reparative processes in the myocardium. Earlier, we demonstrated that urokinase expression significantly increased immediately after simulation of myocardial infarction in rats, but after a few days, it dropped below the baseline level in an unaffected myocardium (unpublished data). This suggested that an increase in urokinase expression in the heart following myocardial infarction may stimulate reparative processes through activation of growth factors. To test this suggestion, we used plasmid expression of urokinase in the peri-infarction area of the rat’s heart, which promoted significant stimulation of the reparative/regenerative processes in the heart: neovascularization and a decrease in the size of infarction and post-infarction fibrosis [151]. These results indirectly indicate the involvement of urokinase in heart recovery after myocardial infarction; however, the mechanisms of this involvement have not yet been identified.

The main trigger initiating post-infarction remodeling is known to be death of cardiomyocytes, which is accompanied by the development of an aseptic inflammatory reaction, redistribution of extracellular matrix proteins, and recruitment of stem/progenitor cells to the damaged area. Along with other components of the extracellular matrix, vitronectin is involved in this process. However, unlike most of these proteins synthesized by heart cells, vitronectin is formed mainly in the liver, where from it enters gets into the systemic circulation and then accumulates in the damaged area. We demonstrated that vitronectin was almost completely absent in the intact myocardium, but its level increased significantly after the experimental myocardial infarction, and the dynamics of its accumulation correlated with accumulation of heart progenitor cells (HPCs) in the infarction and peri-infarction areas. Earlier, using immunohistochemical staining, we showed that the urokinase receptor was present on the surface of HPCs in the myocardium; the receptor remained during cultivation of HPCs *in vitro *and was able to specifically bind vitronectin [152, 153]. Furthermore, HPCs isolated from the myocardium of *uPAR *knockout mice much poorly adhered to vitronectin than HPCs derived from the heart of wild-type mice (HPCs^WT^). In addition, inhibition of the urokinase receptor by specific antibodies on the surface of HPCs^WT^ led to a decrease in the ability of cells to adhere and spread on the vitronectin matrix [152]. Therefore, we suggested that uPAR may act as a regulator of the adhesive properties of HPCs, which may become a determining factor in their accumulation and integration within the damaged area. The interaction between uPAR and vitronectin can be either independent of integrins or be due to the activation of various integrins [154], thereby modulating the choice of the matrix for interaction [155-157]. Elucidating the role of uPAR and other components of the urokinase system in the regulation of the epithelial-mesenchymal transition of epicardial cells and the mechanisms of their participation in the regulation of the interaction of HPCs with various extracellular matrix proteins, their migration, and proliferative and differentiating properties is the object of our further research.

## CONCLUSION

**Fig. 3:**
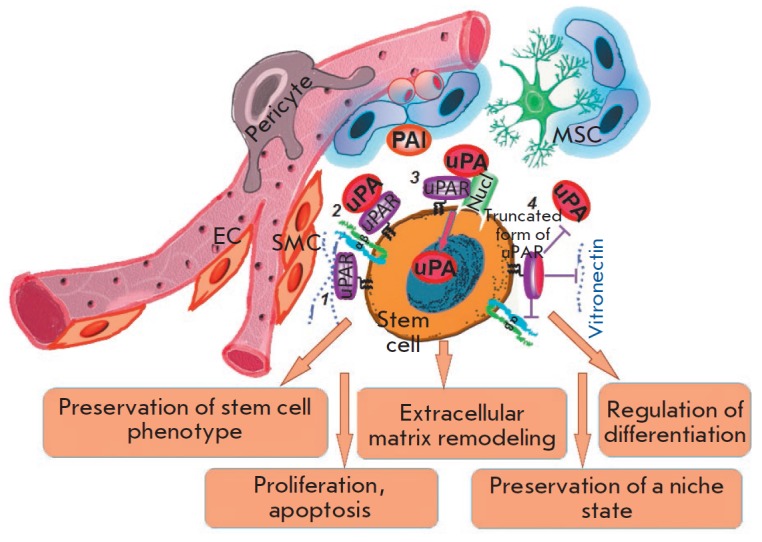
The urokinase system modulates the state of stem cells in cell niches. The interaction between urokinase and the urokinase receptor promotes localization of the proteolytic activity on the cell surface, which, in turn, leads to the extracellular matrix remodeling necessary for maintaining the microenvironment of the cell niche. In addition to active participation in proteolysis, the urokinase–receptor complex (*1, 2*) interacts with vitronectin, an important extracellular matrix protein, and is able to co-localize with integrins, growth factor receptors, and other molecules inside the signaling complex, which leads to activation of intracellular signaling and, as a result, to preservation of the stem cell phenotype, as well as to regulation of proliferation/apoptosis and differentiation. Urokinase proteolytic activity is regulated by inhibitors of plasminogen activators, PAI-1 and PAI-2. With participation of nucleolin, urokinase can be transported into the nucleus (*3*), which can lead to activation of a unique self-sustaining program or, conversely, to reduced adhesion, escape of cells from the niche, and migration to the damaged area. The urokinase receptor can be proteolytically cleaved by various molecules (*4*), which inhibits its ability to bind ligands (uPA and vitronectin), interact with integrins, and activate the appropriate signaling mechanisms. SMC is a smooth muscle cell

**Fig. 4:**
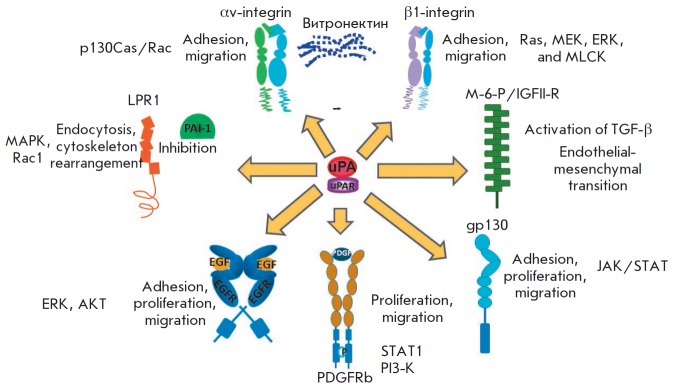
Main signaling molecules involved in urokinase system activity

**Fig. 5:**
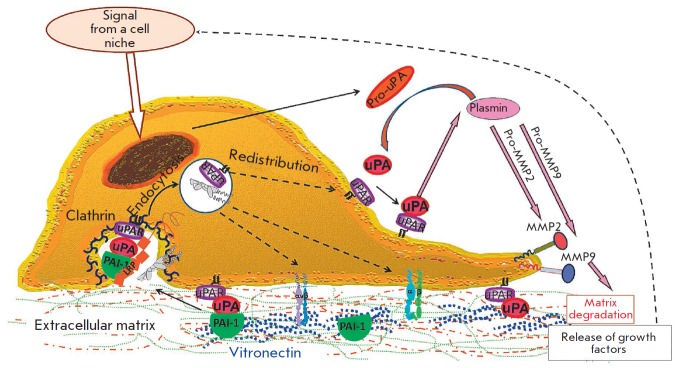
Involvement of the urokinase system in the regulation of stem/progenitor cell migration. Specific signals arising in the cell niche promote the formation of a promigratory phenotype of the stem/progenitor cell and an increase in the production of urokinase, its receptor, and other factors necessary for cell migration. Urokinase activates a proteolytic cascade involving plasminogen and matrix metalloproteinases (MMPs). This leads to cleavage of the extracellular matrix and release of latent growth factors and PAI-1. PAI-1 inactivates urokinase, and a newly formed complex acquires high affinity for LRP-1 that mediates clathrin-dependent endocytosis. This triggers intracellular signaling, cytoskeleton rearrangement, and redistribution of the urokinase receptor at the leading edge of the cell, which promotes directional migration

The stem cells of an adult organism exist in a set microenvironment, the so-called cell niches, that controls their ability to self-renew and the level of proliferation and differentiation. In niches, stem cells occur in close connection with committed progenitor cells, stromal cells, and extracellular matrix proteins the interaction with which regulates maintenance of the resting state, optimal metabolic profile, and low differentiated state, as well as processes of differentiation and release of stem cells from the niche after reception of an appropriate stimulus. Numerous studies suggest that the urokinase system coordinates specific signals from the components of the extracellular matrix and surrounding cells (*Fig. 3*). Its main components (uPAR and uPA) are abundant in the cells that form tissue cell niches, including stem cells and microenvironment cells, and their suppression in most cases leads to decreased proliferation, transition of stem cells to the resting state, induction of apoptosis, and inhibition of invasion, migration, and differentiation. Inhibitors of plasminogen activators regulate the functions of stem/progenitor cells by limiting extracellular proteolysis to ensure specialized functions for progenitor cells, as well as maintaining the competitive interaction of vitronectin with integrins and uPAR and recirculation of uPAR on the cell surface. The influence of urokinase system components on stem cell functions is associated with both differential regulation of the activity of a big variety of signaling molecules (*Fig. 4*) and direct action of urokinase in the nucleus, which may induce a unique program of stem cell self-maintenance or, conversely, lead to reduced adhesion, escape of cells from the niche, and activation of their migration to the damaged area (*Fig. 5*). The main role in this process is apparently played by the urokinase receptor that represents a part of a large signaling complex consisting of a variety of proteins, both outside and inside the cell, which triggers intracellular signaling. One can suggest that the uPAR composition and its interaction with various partners represents an evolutionarily conservative key that determines the molecular features and retention of stem cells in the cell niche. To this end, uPAR functions are modulated by proteolytic cleavage, which leads to the formation of truncated membrane-bound forms of uPAR (c-uPAR), as well as soluble forms of the urokinase receptor (su-uPAR). c-uPAR lacking the D1 domain cannot bind ligands (uPA and vitronectin), interact with integrins, and activate the appropriate signaling mechanisms. In addition, the soluble form su-uPAR can compete with the membrane-bound form uPAR for binding to ligands, thereby limiting signal transduction into the cell, extracellular proteolysis, and adhesion. This highly controlled system which regulates location and functions of stem cells in cell niches opens up new opportunities for the development of approaches to specifically regulate their differentiation and other functions. Elucidation of the mechanisms maintaining the balance of proliferation/ apoptosis, migration, and differentiation of the stem cells controlled by urokinase system components is an important biological and medical problem that should be resolved as soon as possible. Targeting the uPA/ PAI/uPAR system alone or in combination with other signaling pathways may hold promise in improving the therapeutic potential of stem/progenitor cells or helping eliminate tumor stem cells during treatment of cancer diseases.
